# Implementation and Evaluation of the Clear Dx Platform for Sequencing SARS-CoV-2 Genomes in a Public Health Laboratory

**DOI:** 10.1128/spectrum.04957-22

**Published:** 2023-03-28

**Authors:** Arunachalam Ramaiah, Manjeet Khubbar, Samantha Scott, Amy Bauer, Jennifer Lentz, Katherine Akinyemi, Addie Skillman, Joshua Weiner, Nandhakumar Balakrishnan, Sanjib Bhattacharyya

**Affiliations:** a City of Milwaukee Health Department, Milwaukee, Wisconsin, USA; b Georgia Public Health Laboratory, Decatur, Georgia, USA; Emory University School of Medicine

**Keywords:** SARS-CoV-2, COVID-19, Clear Dx, whole-genome sequencing, genomic surveillance

## LETTER

Severe Acute Respiratory Syndrome Coronavirus 2 (SARS-CoV-2) is an etiological agent of the ongoing coronavirus disease 2019 (COVID-19) pandemic, which has infected over 750 million people globally, with 1% mortality rate (World Health Organization, accessed 8 February 2023) (1). During this enormous human health crisis, whole-genome sequencing (WGS) has enabled public health agencies to identify circulating SARS-CoV-2 variants, understand vaccine breakthroughs and transmission patterns, and contact tracing investigations (2, 3). WGS involves library preparation, sequencing, and bioinformatics data analysis. However, manual library preparation and data analysis are immensely labor-intensive processes that likely increase workflow errors and reduce the consistency of results. This may have resulted in significant delays to real-time SARS-CoV-2 genomic surveillance and monitoring of the variants for public health action.

**FIG 1:**
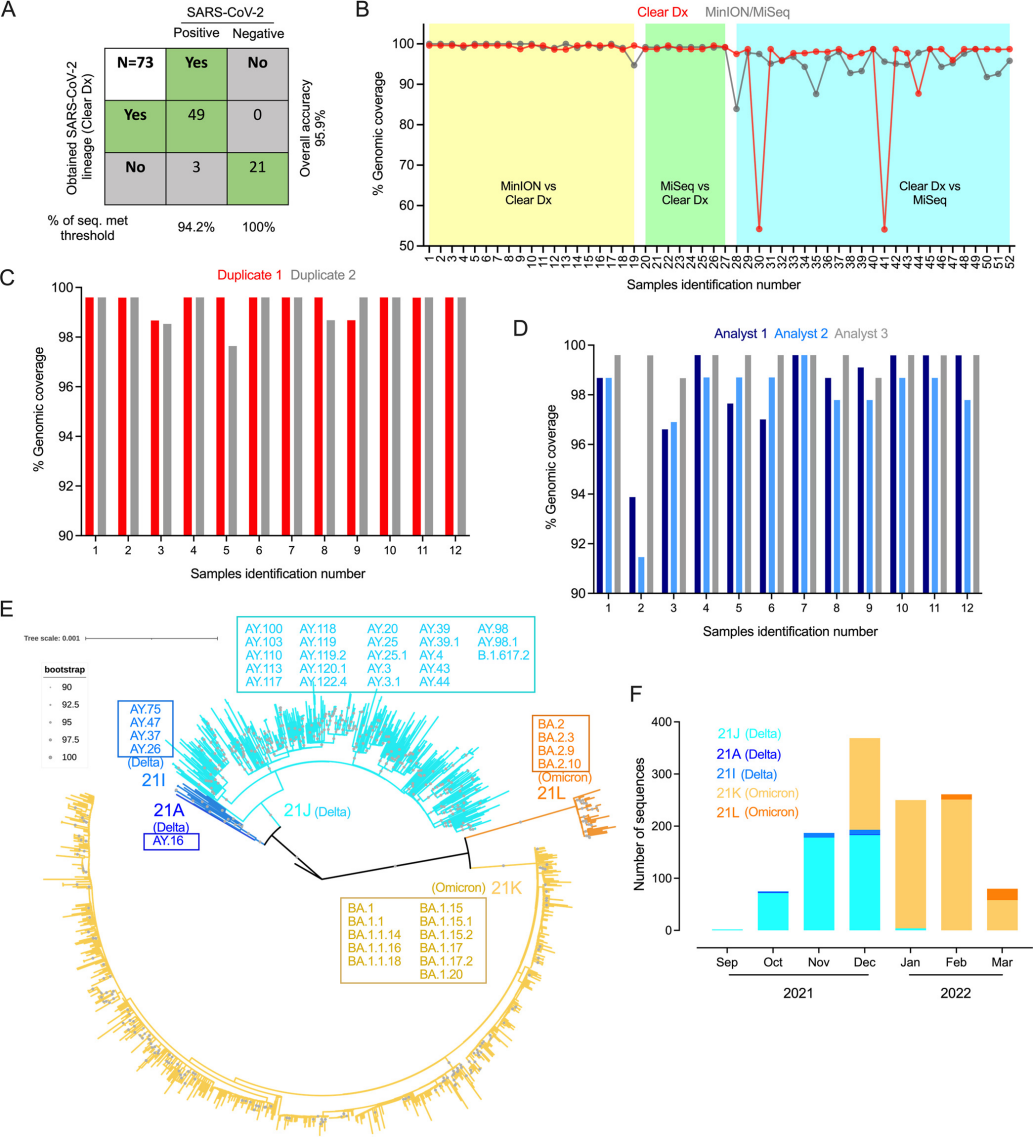
Accuracy and precision of Clear Dx platform on SARS-CoV-2 genomic surveillance. (A) A total of 73 specimens comprising 52 SARS-CoV-2-positive clinical specimens previously sequenced at MHDL either on MinION or Illumina MiSeq (second column), and 21 SARS-CoV-2 negative specimens (third column) were used for Clear Dx validation. (B) Genomic coverage (%) of 52 SARS-CoV-2-positive specimens used to study the performance characteristics of the Clear Dx platform for SARS-CoV-2. Previously sequenced 27 of 52 SARS-CoV-2 specimens either on the MinION (*x* axis sample numbers 1 to 19, yellow background) or MiSeq (20 to 27, green) platform, and then verified using the Clear Dx platform. The remaining 25 of 52 SARS-CoV-2 samples were sequenced initially using Clear Dx and subsequently verified using the MiSeq platform (28 to 52, blue). The positive and negative predictive results of these 73 samples were used to calculate sensitivity and specificity of Clear Dx. For precision testing, (C) the repeatability was measured by testing previously sequenced 12 SARS-CoV-2-positive samples in duplicate in the same run by a single operator, and (D) the reproducibility was measured by testing these samples separately in three runs in three different days by different operators. (E) The phylogenetic representation of all 1,224 SARS-CoV-2 genomes sequenced from clinical specimens of the populations in Milwaukee, Wisconsin, and nearby counties using Clear Dx. The maximum likelihood circular tree showed two major clades, one consisting of three major clusters, including 21A (Delta), 21I (Delta), and 21J (Delta), and a second one consisting of two clusters, including 21K (Omicron) and 21L (Omicron) clade strains, following the naming convention and branching colors in Nextclade. The corresponding Pango lineages and sublineages for each of these five Nextclades were provided in the box. We displayed bootstrap values, if they are ≥90% supported (gray circles in the middle of the branches). Most of the nodes in the tree were formed with 100% bootstrap supports (large gray circles in the middle of the branches), confirming that the split of the branches was supported with high confidence. The distance corresponding to substitution per site is indicated by a scale bar. (F) Chronologic distribution of SARS-CoV-2 genomic variants for a 7-month period (September 2021 to March 2022) in the population of Milwaukee and nearby counties. The clades for 1,224 SARS-CoV-2 genome sequences were identified in Nextclade and classified based on the sampling date. Clades are color coded following the naming convention and branching colors in Panel E.

Clear Dx (Clear Labs, San Carlos, CA) is a fully automated platform for SARS-CoV-2 detection and genomic surveillance that goes from extracted RNA to bioinformatic data analysis without any human intervention, thereby reducing the analytical errors (4). The City of Milwaukee Health Department Laboratory (MHDL) has verified the performance characteristics of the Clear Dx WGS SARS-CoV-2 assay as recommended by the manufacturer. A total of 75 clinical specimens, such as nasopharyngeal and nasal swabs, comprising 54 SARS-CoV-2-positive specimens and 21 other respiratory viral-pathogen-positive specimens, but negative for SARS-CoV-2 (Table S1 in the supplemental material), from MHDL frozen specimen inventory were included for the verification. Of 54 SARS-CoV-2-positive specimens, 27 were previously sequenced either on the MinION (Oxford Nanopore Technologies, ONT) or MiSeq (Illumina Inc.) platform, while the remaining were sequenced first in Clear Dx and subsequently verified using the MiSeq platform. Two of the samples sequenced in Clear Dx subsequently failed in MiSeq; we therefore continued with the remaining 52 SARS-CoV-2-positive specimens. Based on the verification, 95.9% overall accuracy was obtained with sensitivity of 94.2% (49/52) (in terms of ≥90% genomic and ≥100× sequencing depth/coverages as quality control metrics [QC] of precisely identified SARS-CoV-2 lineage) and specificity of 100% (21/21) (in terms of no amplification and detection of SARS-CoV-2 in the specimens previously positive for other respiratory virus pathogens) (Fig. 1A and B; Table S1). The remaining three (3/52) SARS-CoV-2-positive specimens were assigned accurate lineages with only <90% genomic coverages in Clear Dx (Table S1). The genomic coverage ranges between 54.1 and 99.6% (median, 98.7%; mean, 96.8%) for Clear Dx-generated sequences and 83.9 to 100% (median, 98.9%; mean, 97.3%) for sequences from the other two platforms (Fig. 1B). These results suggest that coverage discrepancies were possibly caused by either an area of low amplification and sequence drop-out due to low performance of ARTIC V3 primer set in that region, or sequencing errors (5). Furthermore, the bases or regions with low amplification possibly caused an erroneous frameshift in the sequence assemblies. A further comparison of the sequences generated on Clear Dx and MinION or MiSeq platforms for 52 specimens identified 0 to 1 mutation difference (Table S1), suggesting that sequences were identical in all regions with coverage, and the same biological conclusions might be drawn irrespective of the sequencing platform and data analysis method.

For precision, 12 previously sequenced SARS-CoV-2-positive specimens that had diverse and descendant lineages with essential QC (in terms of ≥90% genomic and ≥100× sequencing depth/coverage) parameters were selected (Table S2). Repeatability was assessed by testing these specimens in duplicate in the same run by a single operator, and reproducibility was assessed in three separate runs on three different days by multiple operators. Both repeatability and reproducibility of base calling on all these 12 clinical samples met QC requirements, with genome coverage ranges between 97.6 and 99.6% for intra-runs (median, 99.6%; mean, 99.4%) and 91.5 to 99.6% for inter-runs (median, 98.7%; mean, 98.5%), respectively (Fig. 1C and D; Table S2). The sequencing depth ranged from 461× to 11,051× (median, 1,903.5×; mean, 2,692×) for intra- and 211× to 5,433× (median, 664.5×; mean, 1,356×) for interassays. The Nextclade-based analysis confirmed the accuracy in clade assignment for these 12 specimens, indicating the efficiency and stability of Clear Dx in characterizing SARS-CoV-2 genomes.

Between October 2021 and March 2022, an additional 1,440 nasopharyngeal/nasal swabs submitted to MHDL from the City of Milwaukee and surrounding communities were sequenced for SARS-CoV-2 genomes by the Clear Dx platform. Of 1,440 sequenced specimens, 1,224 (85%) of the sequences that fulfilled the established QC metrics were used for further analysis (Table S3) (4). The maximum likelihood phylogeny showed that these sequences were distributed into five clades, in which >95% of the sequences were derived from two clades (21K Omicron, 59.7%; 21J Delta, 35.9%) (Fig. 1E). During this study period, 43 lineages/sublineages belonging to five clades of SARS-CoV-2 were circulating in the local communities (Fig. 1E and F), of which BA.1.1 (22%), AY.103 (13.8%), and BA.1.15 (13.3%) were the most identified sublineages from these specimens (Fig. S1). The overall sequencing lineages from the Clear Dx platform correlate with the Wisconsin SARS-CoV-2 genomic surveillance data (3).

In conclusion, the sequence data generated by Clear Dx automated sequencing platform are being efficiently used for near real-time genomic surveillance, epidemiological investigations, and identifying SARS-CoV-2 variants circulating within the City of Milwaukee jurisdiction. Most importantly, the sequencing turnaround time has significantly decreased from 2 weeks to an average of 5 days from the date of specimen collection. This implementation has also reduced staffing hands-on time, thereby improving workforce capacity assisting other public health testing activities. Though SARS-CoV-2 genome sequencing by Clear Dx platform might be quicker and more user-friendly, the consumables are 1.5- and 2-fold more expensive than the consumables required to manually prepare libraries and sequence them on long-and short-read-based MinION and MiSeq sequencing platforms, respectively, per sample. In the near future, expanding this platform to sequence other emerging and reemerging infectious disease pathogens may potentially assist public health response during an outbreak investigation and surveillance activities.

### Data availability.

All 1,224 SARS-CoV-2 genomes sequenced on the Clear Dx platform have been deposited with the Global Initiative on Sharing All Influenza Data (GISAID, https://www.gisaid.org/). The accession numbers for all these sequences are provided in Table S3.

## References

[1] Huang C, Wang Y, Li X, Ren L, Zhao J, Hu Y, Zhang L, Fan G, Xu J, Gu X, Cheng Z, Yu T, Xia J, Wei Y, Wu W, Xie X, Yin W, Li H, Liu M, Xiao Y, Gao H, Guo L, Xie J, Wang G, Jiang R, Gao Z, Jin Q, Wang J, Cao B. 2020. Clinical features of patients infected with 2019 novel coronavirus in Wuhan, China. Lancet 395:497–506. doi:10.1016/S0140-6736(20)30183-5. 31986264 PMC7159299

[2] Aggarwal D, Page AJ, Schaefer U, Savva GM, Myers R, Volz E, Ellaby N, Platt S, Groves N, Gallagher E, Tumelty NM, Le Viet T, Hughes GJ, Chen C, Turner C, Logan S, Harrison A, Peacock SJ, Chand M, Harrison EM, COVID-19 Genomics UK (COG-UK) Consortium. 2022. Genomic assessment of quarantine measures to prevent SARS-CoV-2 importation and transmission. Nat Commun 13:1012. doi:10.1038/s41467-022-28371-z. 35197443 PMC8866425

[3] Ramaiah A, Khubbar M, Bauer A, Scott S, Lentz J, Akinyemi K, Skillman A, Weiner J, Balakrishnan N, Bhattacharyya S. Genomic surveillance identifies SARS-CoV-2 transmission patterns in local university populations, Wisconsin, USA, 2020–2022. Microb Genom, in press. 10.1099/mgen.0.000970 PMC10132055 37000821

[4] The Clear Dx Rapid, Fully Automated Whole Genome Sequencing Platform for SARS-CoV-2 Detection and Genomic Surveillance. https://www.clearlabs.com/cleardx/.

[5] Davis JJ, Long SW, Christensen PA, Olsen RJ, Olsen R, Shukla M, Subedi S, Stevens R, Muser JM. 2021. Analysis of the ARTIC version 3 and version 4 SARS-CoV-2 primers and their impact on the detection of the G142D amino acid substitution in the spike protein. Microbiol Spectr 9:e01803-21. 34878296 10.1128/Spectrum.01803-21 PMC8653831

