# Supplementary material for: Implementation and Evaluation of the Clear Dx Platform for Sequencing SARS-CoV-2 Genomes in a Public Health Laboratory
Source: Microbiol Spectr. 2023 Mar 28;11(2):e04957-22. doi: 10.1128/spectrum.04957-22 (PMC10100861; doi:10.1128/spectrum.04957-22)
Supplement: Supplemental file 4 — Supplemental material. Download spectrum.04957-22-s0004.pdf, PDF file, 0.2 MB [file spectrum.04957-22-s0004.pdf]

Implementation and Evaluation of Clear Labs Dx Platform for Sequencing SARS-CoV-2 Genomes in a Public Health Laboratory

Arunachalam Ramaiah[a,#,*], Manjeet Khubbar[a,#], Samantha Scott[a], Amy Bauer[a], Jennifer Lentz[a], Katherine Akinyemi[a], Addie Skillman[a], Joshua Weiner[a], Nandhakumar Balakrishnan[a,b], Sanjib Bhattacharyya[a]

Supplemental File

Methods

The Milwaukee Health Department Laboratory (MHDL) has been testing human nasopharyngeal/nasal swab specimens submitted for SARS-CoV-2 testing according to CDC Interim Guidelines for Collecting and Handling of Clinical Specimens for COVID-19 Testing [1]. For all SARS-CoV-2-positive samples included in this study, nucleic acid extractions were performed using FDA EUA-approved automated extraction platforms, such as Maxwell rapid sample concentrator (RSC) platform (Promega, Madison, WI), NucliSens easyMAG, EMAG (bioMérieux, Boxtel, The Netherlands), EZ1 Advanced XL (Qiagen, Valencia, CA), and KingFisher Flex platforms (Thermo Fisher Scientific, Waltham, MA) as per manufacturer's instructions. The extracts were stored at −80°C until testing [2,3]. Clear Labs Dx WGS SARS-CoV-2 Test is an automated whole-genome sequencing platform for SARS-CoV-2. The workflow begins from extracted RNA to automated library preparation, sequencing and generation of compressed FASTQ and FASTA files with minimal human intervention. The reagent preparation, assay processing, and analysis were performed according to the Clear Labs Dx WGS SARS-CoV-2 manufacturer's instructions and MHDL standard operating procedures. The Clear Labs Dx WGS SARS-CoV-2 Test utilizes pre-packaged reagents and kits. These kits contain different pre-packaged and metered reagents, such as primers, enzyme mixes, buffers, Solid Phase Reversible Immobilization (SPRI) beads, and sequencing specific reagents. All reagents were sealed with pierceable foil that was pierced by pipette tips during the run. The system uses a Hamilton STAR robotic platform for automation of liquid handling and includes required ancillary equipment, such as Hamilton thermal cyclers, a barcode reader, magnet block, and two MinION

nanopore sequencers from Oxford Nanopore Technologies. Total nucleic acids extracted from SARS-CoV-2-positive specimens were amplified using ARTIC V3 (biopipeline BIP-Wv6) or MIDNIGHT (biopipeline BIP-Wv7) primer pools. For each sequencing run, 30 SARS-CoV-2 RNA extracts that met quality control requirements were sequenced as per manufacturer guidelines. Synthetic SARS-CoV-2 RNA controls procured from Twist Biosciences (Twist Biosciences, San Francisco, CA) were included as a positive control. Barcode classification of sequencing reads and assembly of the consensus SARS-CoV-2 genome were automatically performed using a modified version of the ARTIC bioinformatics pipeline (BIP-WV6 or BIP-WV7) in the Clear Labs WGS App (https://wgs.app.clearlabs.com/) and Medaka via ARTIC 1.2.1 in Terra platform (https://terra.bio/). All consensus sequences generated and related metadata for the SARS-CoV-2-positive samples were submitted to Global Initiative on Sharing All Influenza Data (GISAID; https://www.gisaid.org/) (Table S3) [4].

To assess the overall accuracy of the Clear Labs Dx platform, we compared the sequence data generated from the same SARS-CoV-2-positive specimens on MinION or MiSeq sequencing platforms [5,6]. The detailed procedures for manual library preparation, sequencing, and data analysis for these two platforms were described elsewhere [7]. In brief, the ARTIC network workflow was used for the MinION platform. The samples were first converted into cDNA, then a PCR tiling protocol was performed to amplify overlapping 'tiled' sections covering the SARS-CoV-2 genome using V3 primer pools. It yielded tiled 400 bp amplicons as per ARTIC multiplex PCR protocol [8]. After cDNA amplification, end-repair was performed using NEB Next Ultra II End Repair/dA-Tailing Module (New England BioLabs), the amplicons were barcoded for sequencing with the Native Barcoding Expansion pack to prepare the samples for sequencing in a single run and then pooled. A different native barcode ligates to each sample to be sequenced in multiplex. This was followed by ligating sequencing adapters to pooled barcoded samples before the library was prepared for Nanopore sequencing. Samples were sequenced in multiplex on MinION flow cells using either a MinION Mk1B or MinION Mk1C device. Using MinKNOW software v4.1.22, base calling and demultiplexing of reads were performed by following the EPI2ME Labs ARTIC SARS-CoV-2 workflow to produce FASTQ files (https://github.com/epi2me-labs/wf-artic/). Raw reads in FASTQ format were processed using the ARTIC bioinformatics pipeline (https://github.com/artic-network/artic-ncov2019) [9], where the demultiplexed raw FASTQ files were mapped to the reference virus genome Wuhan-Hu-1 using minimap2 [10] to generate the consensus sequences and variant calls using Medaka.

Using the Illumina DNA Prep library kit on MiSeq platform [6], we sequenced SARS-CoV-2 genome by following the sample preparation procedures used for MinION from converting RNA into cDNA, PCR tiling, primer selection to amplification of targeted regions. Subsequently, sequencing libraries were prepared by adding specialized adapters to both ends of amplicons by tagmentation chemistry. These adapters contain complementary sequences that allow the DNA fragments to bind to the flow cell. Subsequently, these fragments were amplified and purified. The multiplex libraries prepared were pooled together and sequenced. During adapter ligation, unique index sequences, or barcodes were added to each library. During data analysis, these barcodes were used to distinguish the libraries. The FASTQ files generated from the MiSeq platform were assembled using Illumina DRAGEN COVID Lineage (v3.5.4 to v3.5.9), where the reads aligned to a reference genome for calling variants and producing a consensus genome sequence for each specimen.

The clade and lineage assignment for SARS-CoV-2 genomes were uniformly performed using Nextclade Pango Classifier on Nextclade version web-2.8.1 [11]. For the phylogenetic analysis, all 1,224 SARS-CoV-2 genomes along with a reference Wuhan-Hu-1/2019 (GenBank # MN908947) genome sequences were aligned using MAFFT v.7.505 [12]. Subsequently, aligned sequences were used to identify GTR+F+R2 as a best-fit model based on the Bayesian Information Criteria using ModelFinder [13]. The maximum likelihood (ML) phylogenetic tree of SARS-CoV-2 was constructed with 1,000 bootstrap replicates in IQ-TREE multi core version 2.0.3 [14]. The phylogenetic tree was visualized in Interactive Tree Of Life (iTOL) [15]. Prism GraphPad v9.4.1 (www.graphpad.com) was used for generating figures.

Ethical Statement

All specimens or nucleic acid specimens used in this study were obtained under Milwaukee Health Department (MHD) Institutional Biosafety Committee (IBC) guidelines, and per MHD Institutional Review Board (IRB) and Ethics Committee approval. MHD IRB and ethics committee determined the laboratory analysis and Genomic epidemiological activities were for non-research public health surveillance and had no ethical concerns**.**

References

1. Centers for Disease Control and Prevention. 2021. Interim guidelines for collecting and handling of clinical specimens for COVID-19 testing. Summary of recent changes. Key

points. Collecting and handling specimens safely. https://www.cdc.gov/coronavirus/2019-nCoV/lab/guidelines-clinical-specimens.html

2. Centers for Disease Control and Prevention. 2020. CDC 2019-novel corona-virus (2019-NCoV) real-time RT-PCR diagnostic panel. For emergency use only. Instructions for use. https://www.fda.gov/media/134922/download. Accessed 14 March 2021
3. Centers for Disease Control and Prevention. 2020. CDC influenza SARS-CoV-2 (flu SC2) multiplex assay. For emergency use only. Instructions for use. https://www.fda.gov/media/139743/download
4. Khare S, Gurry C, Freitas L, Schultz MB, Bach G, Diallo A, Akite N, Ho J, Lee RT, Yeo W, Curation Team GC, Maurer-Stroh S. 2021. GISAID's Role in Pandemic Response. China CDC Wkly 3(49):1049-1051. doi: 10.46234/ccdcw2021.255.
5. Quick J. 2020. ARCTIC Network amplicon sequencing protocol for MinION for SARS-CoV-2 v3 https://www.protocols.io/view/ ncov-2019-sequencing-protocol-v3-locost-bh42j8ye
6. SARS-CoV-2 Sequencing on Illumina MiSeq Using ARTIC Protocol: Part 2 - Illumina DNA Prep Protocol V.1. 2021. https://www.protocols.io/view/ sars-cov-2-sequencing-on-illumina-miseq-using-arti-n92ld9w1xg5b/ v1
7. Ramaiah A, Khubbar M, Bauer A, Scott S, Lentz J, Akinyemi K, Skillman A, Weiner J, Balakrishnan N, Bhattacharyya S. 2023. Genomic surveillance identifies SARS-CoV-2 transmission patterns in local university populations, Wisconsin, USA, 2020-2022. Microb Genom (In Press).
8. SARS-CoV-2 Sequencing on Illumina MiSeq Using ARTIC Protocol: Part 2 – Illumina DNA Flex Protocol V.1 – Joel Sevinsky, StaPH-B Consortium, Coronavirus Method Development Community. https://www.protocols.io/private/EF7D7A9B84D611EAAB080242 AC110005?step=4
9. Moreno GK, Braun KM, Riemersma KK, Martin MA, Halfmann PJ, Crooks CM, Prall T, Baker D, Baczenas JJ Heffron AS, Ramuta M, Khubbar M, Weiler AM, Accola MA, Rehrauer WM, O'Connor SL, Safdar N, Pepperell CS, Dasu T, Bhattacharyya S, Kawaoka Y, Koelle K, O'Connor DH, Friedrich TC. 2020. Revealing fine-scale spatiotemporal differences in SARSCoV-2 introduction and spread. Nat Commun 11:5558. https://doi.org/10.1038/s41467-020-19346-z
10. Li H. 2021. New strategies to improve minimap2 alignment accuracy. Bioinformatics 37:4572–4574. https://doi.org/10.1093/bioinformatics/btab705

11. Aksamentov I, Roemer C, Hodcroft EB, Neher RA. 2021. Nextclade: clade assignment, mutation calling and quality control for viral genomes. J Open Source Softw 6(67):3773. https://doi.org/10.21105/joss.03773
12. Katoh K, Rozewicki J, Yamada KD. 2019. MAFFT online service: multiple sequence alignment, interactive sequence choice and visualization. Brief Bioinform 20(4):1160-1166. doi: 10.1093/bib/bbx108.
13. Kalyaanamoorthy S, Minh BQ, Wong TKF, von Haeseler A, Jermiin LS. 2017. ModelFinder: Fast model selection for accurate phylogenetic estimates. Nat Methods 14:587-589. https://doi.org/10.1038/nmeth.4285
14. Minh BQ, Schmidt HA, Chernomor O, Schrempf D, Woodhams MD, von Haeseler A, Lanfear R. 2020. IQ-TREE 2: New models and efficient methods for phylogenetic inference in the genomic era. Mol Biol Evol 37:1530-1534. https://doi.org/10.1093/molbev/msaa015
15. Letunic I, Bork P. 2021. Interactive Tree Of Life (iTOL) v5: an online tool for phylogenetic tree display and annotation. Nucleic Acids Res 49:W1:W293–W296. https://doi.org/10.1093/nar/gkab301

Figure legend and Table captions

Figure S1. Distribution of 1,224 SARS-CoV-2 genomic sequences into 43 lineages/sub-lineages.

Table S1. Overview of 52 SARS-CoV-2 and 21 other respiratory viral pathogens positive specimens used to study the performance characteristics of Clear Labs Dx SARS-CoV-2 Test. A) Twenty seven of 52 SARS-CoV-2 specimens previously sequenced either on MinION or MiSeq, and submitted to the GISAID database. B) The remaining 25 of 52 SARS-CoV-2 samples were sequenced first using ClearLabs Dx and then confirmed using MiSeq platform. C) Details of the 21 other respiratory viral pathogen positive specimens, but negative for SARS-CoV-2. Refer to xlsx file.

Table S2. Overall precision of ClearLabs Dx platform. Previously sequenced 12 SARS-CoV-2 positive samples either on MinION or MiSeq platform were used to determine the repeatability and reproducibility. A) The repeatability was measured by testing these samples in duplicate in the same run by a single operator. B) The reproducibility was assessed by testing these samples in three separate runs on three different days and by different operators. Refer to xlsx file.

Table S3. GISAID identification number and Nextclade lineages for 1,224 SARS-CoV-2 genomes sequenced from nasopharyngeal/nasal swab specimens. Refer to xlsx file.

Figure S1

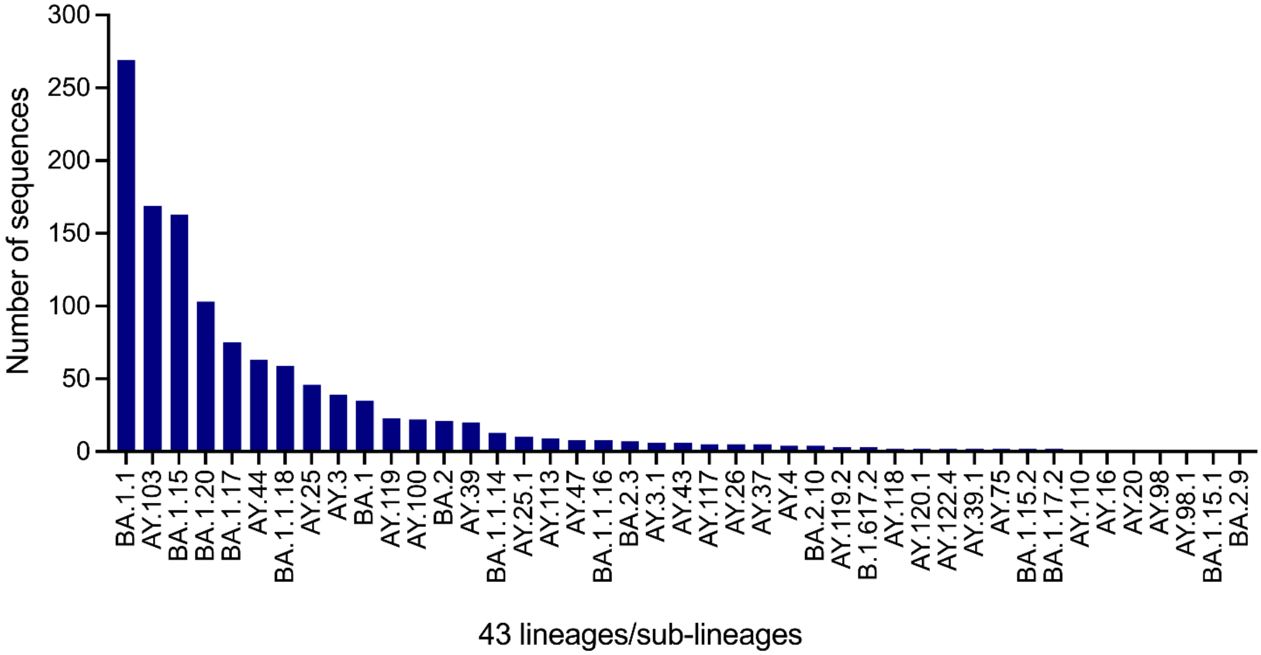

Number of sequences
0
50
100
150
200
250
300
BA.1.1
AY.103
BA.1.15
BA.1.20
BA.1.17
AY.44
BA.1.1.18
AY.25
AY.3
BA.1
AY.119
AY.100
BA.2
AY.39
BA.1.1.14
AY.25.1
AY.113
AY.47
BA.1.1.16
BA.2.3
AY.3.1
AY.43
AY.117
AY.26
AY.37
AY.4
BA.2.10
AY.119.2
B.1.617.2
AY.118
AY.120.1
AY.122.4
AY.39.1
AY.75
BA.1.15.2
BA.1.17.2
AY.110
AY.16
AY.20
AY.98
AY.98.1
BA.1.15.1
BA.2.9
43 lineages/sub-lineages
